# Opening up the ‘black-box’: what strategies do community mental health workers use to address the social dimensions of mental health?

**DOI:** 10.1007/s00127-023-02582-1

**Published:** 2024-01-23

**Authors:** Sumeet Jain, Pooja Pillai, Kaaren Mathias

**Affiliations:** 1 The University of Edinburgh, Edinburgh, Scotland, UK; 2 Herbertpur Christian Hospital, Emmanuel Hospital Association, Dehradun, Uttarakhand India; 3 The University of Canterbury, Christ Church, New Zealand

**Keywords:** Community mental health, Global mental health, Community health workers, India, Social mental health

## Abstract

**Purpose:**

Community-based workers promote mental health in communities. Recent literature has called for more attention to the ways they operate and the strategies used. For example, how do they translate biomedical concepts into frameworks that are acceptable and accessible to communities? How do micro-innovations lead to positive mental health outcomes, including social inclusion and recovery? The aim of this study was to examine the types of skills and strategies to address social dimensions of mental health used by community health workers (CHWs) working together with people with psychosocial disability (PPSD) in urban north India.

**Methods:**

We interviewed CHWs (n = 46) about their registered PPSD who were randomly selected from 1000 people registered with a local non-profit community mental health provider. Notes taken during interviews were cross-checked with audio recordings and coded and analyzed thematically.

**Results:**

CHWs displayed social, cultural, and psychological skills in forming trusting relationships and in-depth knowledge of the context of their client's lives and family dynamics. They used this information to analyze political, social, and economic factors influencing mental health for the client and their family members. The diverse range of analysis and intervention skills of community health workers built on contextual knowledge to implement micro-innovations in a be-spoke way, applying these to the local ecology of people with psychosocial disabilities (PPSD). These approaches contributed to addressing the social and structural determinants that shaped the mental health of PPSD.

**Conclusion:**

Community health workers (CHWs) in this study addressed social aspects of mental health, individually, and by engaging with wider structural factors. The micro-innovations of CHWs are dependent on non-linear elements, including local knowledge, time, and relationships. Global mental health requires further attentive qualitative research to consider how these, and other factors shape the work of CHWs in different locales to inform locally appropriate mental health care.

## Introduction

Since the new millennium, community health workers (CHW) who live in the communities where they work are increasingly recognised as central in health systems [34]. The World Health Organisation defines CHWs as health workers who have received some training (up to 2 years) but are not considered health professionals, and who are based in communities, meaning they provide services outside of health facilities or at peripheral facilities not staffed by health professionals [43]. Decision makers recognise the urgent need to address shortages of health workers, their contribution as service providers working and living in remote and underserved areas and their ability to provide culturally appropriate primary care as well as long-term sustainability [41]. Globally health workers’ rise is linked with the investment of private, bilateral and multi-lateral funders seeking to meet the sustainability development goals (SDGs) [40]. Although CHW roles traditionally have had a focus on maternal and child health, in the past two decades they have expanded to a more comprehensive approach and CHWs now also engage with infectious diseases (like HIV and TB), and the growing burden of chronic illness including mental ill-health [33, 40].

India has one of the largest CHW programmes in the world with approximately 1 million female Accredited Social Health Activists (ASHAs) as key workers within the national health mission. The ASHA programme was launched in 2005 as a key component of the National Rural Health Mission to strengthen rural health service delivery as well as community engagement and ownership and it expanded further in 2015 to include urban areas [41]. The contribution of ASHA workers has also expanded, and they were central in India’s COVID-19 response in the health systems of both rural and marginalised urban communities. This expanding role has also brought growing recognition of ASHAs’ multiple responsibilities and heavy work burden accompanied by poor remuneration [24, 38]. Although the ASHA role has a focus on maternal and child health, this has expanded to include non-communicable diseases and there is growing recognition of their potential to contribute to the care of people with psychosocial disability (PPSD) although this is not currently widespread [42].

The launch of the global mental health movement in a Lancet series in 2007, identified task-shifting as a key role of CHWs to address the shortage of professional human resources. Several studies have demonstrated the importance of community-based workers to promote mental health in communities [12–14, 35] and particularly, in delivering psychosocial interventions, for example, in the Problem Management Plus intervention where CHWs delivering a structured and locally accessible psychosocial intervention [20].

Yet while CHWs have been assigned responsibilities to enable mental health ‘task-shifting’ in low- and middle-income countries, there are challenges linked to this policy solution [1]. These include mental health care responsibilities being added on to a long list of other health system responsibilities, a lack of supervision and training, poor remuneration, the biomedical hierarchy and other asymmetries of power relations linked to CHWs being almost exclusively female in predominantly patriarchal settings [1, 33]. Recent ethnographic literature seeks to unpack the social dynamics of CHWs roles in mental health. For example, both Lang [25] and Kottai and Ranganathan [23] examine the processes through which CHWs perpetuate medicalization in communities in different parts of Kerala, India. Chase [11] working in Nepal, attempts to untangle the different roles (family members, neighbours, health workers) that CHWs hold, arguing that the work of CHWs shifts the very nature of relatedness.

The relationship between structural factors like poor housing, inequalities and poor mental health has received greater attention in recent global mental health publications [6, 36]. Similarly, the importance of mental health as a human right, which is supported by the right to other social determinants, has also risen in prominence in the past five years [8, 37]. However, many global mental health interventions and innovations have failed to address the social determinants that continue to cause poor mental health in the long term [6, 15, 27, 36, 39]. Task-shifting interventions, largely implemented by CHWs, have addressed socio-relational dynamics linked with mental distress (such as isolation or stigma), yet have not addressed social and structural determinants of poor mental health (such as gender inequality and racism). In fact, many CHW programmes have been recognised as perpetuating patriarchal gender relations by giving minimal remuneration, continuously expanding responsibilities of CHW and requiring them to work in settings with little support or training [10, 11, 17]. They also have not sought to support and develop enabling environments which can sustain long-term mental health and wellbeing [6, 27, 36] and have not responded to the felt needs of communities which have led to a low demand for treatment and care [39].

Existing research demonstrates how the work of the ‘social’ is implicit in the practice of CHWs who use local and contextual knowledge to address risk factors for poor mental health [11, 25], However, these actions are often implicit and may only be found in the hidden ‘nooks’ and ‘crannies’ of programmes [5, 38]. A recent review identified that when CHWs can play a role beyond service delivery to address upstream causes of ill health, they have the potential to catalyse ‘social, political and health system transformation’ [2]. Identifying how and where contexts and structural determinants are addressed in both micro and macro ways is important to the field of global mental health as it moves from a biomedical understanding of treatment ‘gaps’ used at its inception to a broader, de-colonial, trans-disciplinary, trans-diagnostic approach which engages with assets, and local and contextual perspectives [5].

Maes [28] advocates for the importance of studying the interactions of CHWs with various actors in health systems, including clients, as a means to understand the impact of CHWs on health and wellbeing. In contextualizing our findings, this paper engages with published studies that examine the ways that CHWs use their local and contextually informed understanding of social and structural health determinants, to promote and support psychosocial wellbeing and mental health (e.g. [9, 25]). We argue that greater attention should be given to the ways that CHWs operate and the strategies and mechanisms they deploy to improve mental health (following [2, 11, 25]). An important issue raised by Lang [25] in the Kerala context is the processes through which CHWs translate between biomedical concepts and social and cultural frameworks that are acceptable and accessible to community members. Existing work also suggests a nuanced understanding of what CHWs do in delivering mental health care, will have implications for understanding their role in positive mental health outcomes, such as social inclusion and recovery. However, the specifics of how CHW activities in mental health care might shape outcomes has had limited consideration in the literature. Our study examines the types of skills and strategies to address social dimensions of mental health used by community health workers (CHWs) working together with PPSD in a non-governmental project in north India. Unlike earlier ethnographic studies [9, 11, 23, 25] we draw primarily on interviews with CHWs, secondary data (case notes, care plans) and limited observation. This allows us to also develop initial insights into (mental health) outcomes of CHW interventions.

## Methods

### Setting

This study was set in bustling peri-urban settings of Dehradun, the capital city of the north Indian state of Uttarakhand. This sprawling periurban district has 1.96 million residents. The dominant patriarchy is evidenced in the sex ratio of 902 females born to every 1000 males while the national sex ratio is 940. Disadvantage for women is further evident in the disparate literacy rates which are 78% for women and 89% for men [18].

It was hosted by Burans, a non-profit community mental health partnership project that started in 2014 (www.burans.org). The three geographical communities engaged in this study were: (1) Sahaspur, a town of 100,000 people living in closely co-located and informal brick housing; (2) Kanwali road, in the centre of Dehradun city, where 80,000 people lived in informal housing, lining a large open sewer; and (3) Mussoorie, a tourist town one hour’s drive from Dehradun, where a large service-industry supports hotels, with 35,000 residents (insert map indicating these areas).

In each community, five employed team members worked with community members to promote mental health through increased knowledge, safe social spaces and partnerships for action [7, 29] and by strengthening the public mental health system. Burans works with community members who self-identify as experiencing mental distress and who are referred to as people with psychosocial disability (PPSD), which is the term preferred by people with lived experience of mental health problems in India [30]. People describing anxiety, unexplained somatic symptoms and/or depression who were able to fulfil most daily responsibilities were categorised as having a common mental disorder (CMD). People (or their caregivers) describing the loss of social networks, lack of self-care and/or auditory hallucinations or delusions and unable to fulfil their daily responsibilities were assessed as having a severe mental disorder (SMD). The majority of PPSD experience common mental health problems such as anxiety and depression and do not have access to biomedical care and do not have a formal diagnosis. Further detail of the types of problems experienced and demographics of PPSD within the Burans programme are described elsewhere [30, 32]. The CHWs working with Burans are women from the local informal urban community, who have completed high schooling and have shown evidence of engaging in their neighbourhood as change agents. Each CHW delivers a mix of individual psychosocial support to PPSD following the structured Burans ‘care plan’ which uses a coproduced recovery tool, Swasthya Labh Sadhan to support PPSD to take active steps to engage in eight domains of recovery [31]. CHWs also use validated psychometric scales to measure social participation and mental health outcomes, which have been documented in a prospective cohort study [32], CHWs also implement a range of community organisation activities as well as group facilitation for young people, caregivers using structured interventions. CHWs also support PPSD to access biomedical care by accompanying them to the Government psychiatry clinic for the first time, CHWs are trained and coached twice weekly by a social worker-trained team leader, who supports four to five CHWs. Inception training is 15 days spread over four months and includes modules in community organisation, group facilitation as well as technical topics related to mental health, active listening and problem solving and other psychosocial skills as well as advocacy and access to health care. They work eight hours a day and are paid by Burans. A published case study provides further details of the implementation team and their approach [30].

### Data collection

Research participants included both people with psychosocial disability (PPSD) who were registered with the Burans non-profit programme as well as CHWs working with Burans. At the time of this study, there were 1000 PPSD registered with the Burans programme. To ensure we selected a non-biased sample, we used a random number generator to identify 46 PPSD from the electronic register to invite participants in this study. Inclusion criteria were that PPSD were over the age of 17 years and had participated in meetings with the Burans team at least six times. People who were no longer resident of the Dehradun district were excluded. All selected participants consented to participate in this study.

In the second half of 2017, PP and KM interviewed seven of a total of nine CHWs about their PPSD clients using a semi-structured interview format that probed their understanding of the client’s problems, their engagement and responses to PPSD and the barriers they faced. Interviews were conducted in Hindi, and audio-recorded and detailed notes were made from recordings and translated into English. KM and PP as employees in the same organisation as the CHWs also spent several weeks shadowing the CHWs while they went about their work in the community. They sat in on conversations, watched how CHWs interacted and built connections and took written notes describing their observations of CHWs working with PPSD. They also read and reviewed the hand-written care plan records that were kept by CHWs after each home-based visit. The information collected in these care plans is described in greater detail in two published studies [30, 32].

We used a case study analysis approach, considering each CHW as a case study with the three data sources of interview notes, their written care plan records and observations of each CHW in action. Interview data were coded emergently by KM and SJ, paying attention in particular to the strategies used by CHWs and the ways that they identified and engaged with social and structural determinants of mental health.

Limitations were that while interviews were conducted in Hindi, the interviewers (PP and KM) did not speak the local dialect of the communities where PPSD and community workers were based, which may have limited understanding of the context. Additionally, some of the interviews risked social desirability bias as both interviewers were also involved in project operations and they may have been perceived as providing access to resources. The positive aspect of project implementation team members participating in data collection is that they had a strong understanding of the structural factors that contribute to mental health problems and had many interactions and observations of PPSD which could contribute to and enrich their analysis of data.

## Positionality and ethics

The identities of researchers are likely to have influenced the findings both through providing objectivity as well as subjective assumptions. All researchers spoke fluent Hindi as a second language: PP is an Indian national and KM is a New Zealand national, both lived in Dehradun at the time of research; SJ is of Indian and Canadian origin and lives in Scotland.

Signed informed consent was obtained from all PPSD participants, for their care plans to be reviewed and their cases discussed with CHWs. The Institutional Ethics Committee of the Emmanuel Hospital Association, New Delhi, granted approval to the ethics proposal for this study in January 2017.

## Findings

We present our findings first through a case study that illustrates several key themes emerging from the data set. The case study intends to provide a holistic analysis of the interactions between one CHW and one client. We then examine each of these key themes, in turn, drawing on additional examples from our dataset.

### Case study—Priya (CHW) and Rita (PPSD)

Priya was a 24-year-old community worker who had completed 12 years of schooling and lived with her parents in a low-income community two kilometres from the community she worked in as an employed team member. She identified PPSD through door-to-door knocking and meetings in the low-income community adjacent to her home.

Rita was an 18-year-old young Muslim woman who lived with her widowed mother and two brothers in a tiny two-room house in an informal urban community. After her fathers’ death, Rita had been required to leave school after completing 10 years of schooling to tailor clothes in order to generate supplemental income for the family. Although Rita enjoyed tailoring at home, watching her peers go to school meant Rita felt socially isolated, disadvantaged and experienced frequent concerns about the future, and stayed at home without meeting friends and felt lonely. Rita’s mother being the key decision maker after her father’s death, also faced mental health problems, which further exacerbated Rita’s condition. Rita’s status as a young, unmarried Muslim woman restricted her freedom of movement, a common experience for people like her in north India [16].

#### CHW response

Priya visited Rita over a five-month period, and described spending time visiting and talking to Rita on multiple occasions to develop a relationship of mutual trust. She described their conversations to understand what being well would look like to Rita who described feeling lonely and wishing she could take more initiative. Addressing these hopes formed the basis of further responses.

Firstly, increasing opportunity for social connection required that the two women develop a three –step bespoke plan: Priya (CHW) reported that initially Rita felt embarrassed to meet up with others as she felt she could not converse well with her limited education. Further, after several months almost fully at home, Rita lacked social confidence. Priya described encouraging Rita to believe in herself and did this by role-playing peer conversations with Rita. When Rita felt more confident, Priya sought to support Rita to connect more socially by inviting her to join a sewing class in her neighbourhood for young women. Yet it was not so simple. Belonging to a minority religion with restrictions on freedom of movement, significantly reduced opportunities to leave the house for Rita. Priya problem-solved with Rita and together they proposed that Rita and her mother could host the local sewing group in their own household to avoid family disputes about Rita leaving the house without a male relative. This group sewing programme provided an opportunity to meet with peers and to work together while income generating. She could then converse with peers in the sewing class. Thus, Priya devised and adapted a bespoke intervention to support Rita to build new skills, attitudes and relationships and to increase her access to social connections.

Secondly, the CHW supported socio-culturally appropriate opportunities for income generation and further education. Priya described poverty as the key factor which restricted Rita’s access to education and social connections. Priya recognised sewing as a useful and culturally acceptable skill to generate income for young women from Muslim households as it can be done within the home [19]. Priya described that Rita did well with both learning sewing and making friends in the sewing group and participating also built her sense of self-confidence. Thirdly, describing education as a key health determinant, Priya supported Rita to resume education by introducing her to Open Schooling, a home-based correspondence school which would not constrain her from continuing with expected household roles and income generation with sewing. Priya suggested that Rita could study for a qualification which would help her in the long term. Initially, Rita’s mother was reluctant to allow her to pursue further education. But Priya addressed her concerns by including Rita’s mother in a support group, where multiple discussions on mental health knowledge, gender discrimination and the importance of education took place. She played videos such as the mental health episode of ‘Satyamev jayate’ (a popular television talk show) that talks about real-life incidents of women in distress and caregiving for people with mental illnesses on her phone. This helped Rita’s mother realise the importance of education and she allowed Rita to pursue further education.

### Deep knowledge of context and social determinants

Community workers in our sample displayed a high degree of knowledge of the context of their client’s lives and were able to apply this in developing their responses. This included knowledge across a number of domains including both of more intimate spaces, like family dynamics, as well as wider forces such as environmental and social determinants. The knowledge of the context and structural health determinant was described as implicit (‘I just know’) and linked to their own experiences of living in communities that were socioeconomically similar.

The knowledge and analysis of context took place at different levels. For example, on a steamy afternoon in July, KM and Lalita, the CHW went to visit Deepak, a 25-year-old, unmarried man from the Sikh community. Lalita, the CHW squatted in a brick courtyard and gave KM a summary of Deepak’s story and family situation without any reference to notes. Her description engaged an understanding of the family history (two of Deepak’s uncle’s had gone to jail for an alleged murder), educational background (*“He re-did his first grade several times but eventually he lost interest and when he was in his early teens, he started doing labouring work. Now he does any kind of mazdhuri* (daily labouring)* work.”*)*.*

*She could then describe the sources of income for the family and the broader economic situation they faced* (they owed money to multiple relatives and money lenders and were extremely anxious about this). Lalita could thus analyse the factors shaping Deepak’s poor mental health. KM also noted that Lalita knew the names and identities of all the people (grandma, nephew, niece and visiting friend) involved in Deepak’s life.

Lalita also described the story of Deepak’s issues with substance misuse, a problem that affects his father and his two brothers. Her detailed analysis of the family dynamics included descriptions of the impact of the father’s substance misuse on the poor relationships with the sons; and the mother’s relationship with Deepak.

Lalita also reviewed and discussed the outcomes of the psychosocial interventions she had employed with Deepak (psychosocial counselling and support). She described that they did not result in significant improvement in his situation, either in terms of the standardized scores of mental well-being used, or in convincing him to seek rehabilitation but she could reflect critically on possible reasons for this.

After spending time talking with Deepak who roused himself from a dark room off the courtyard, Deepak stated he needed to head out. Back at the office, Lalita continued telling of her analysis of the situation for him. She described that one benefit for Deepak after he joined a four-month psychosocial group intervention was that he engaged with others who had higher status in the community, which she hypothesized, improved Deepak’s social capital and connections. Lalita also suggested that this outcome was tempered by factors that limited Deepak’s ability to address substance misuse which included a circle of friends who sometimes financially supported his substance misuse and the disregard and negative judgement he experienced from his parents.

In this example, the CHW, Lalita, demonstrated how she had applied a nuanced understanding of the links between family history, education, social status and economic disadvantage to engage with the interplay of structural and environmental health determinants.

Pushpa is another CHW who described her work with Sunita and her family in a town on a steep hill. She lived in a house with tarpaulin for roofing, and a dirt floor and in monsoon water ran through her house. Pushpa described how this made it very difficult for Sunita’s children to play and that the wet and dirty house led to worsening mental distress. Pushpa had visited Sunita most weeks at different times of day over a period of four or five months and so she felt she had had a clear idea of the social and economic factors which led to Sunita’s mental distress. Pragmatically, she then supported Sunita and her neighbours to file a Right to Information^1^ request about why there was no water connection in their informal urban community, to prompt action by the local government.

CHWs described multiple ways that they engaged with social health determinants. For example, another CHW described negotiating with the parents of a 17-year-old PPSD client, to encourage them to allow her to attend school alongside her brothers, addressing gender relations. Another CHW described supporting a family with very low income to get access to epilepsy medicines by identifying local Government pharmacies who would provide them with free medication. Multiple CHWs described supporting families to complete the forms and doctors' assessments required in order to receive a disability pension, addressing the right to health.

Another example of a CHW addressing social determinants included engaging in dialogue with people in the neighbourhood to increase awareness about the medical condition of their young neighbour with epilepsy to reduce stigma and name calling.

In-depth knowledge of the context in some instances may have been negative for PPSD. For example, a community worker who knew a client and his family well judged that they were not likely to benefit from psychosocial support based on their family history, describing the criminal activities of his uncle and father. In this instance, family knowledge potentially led to prejudice and reduced psychosocial support.

### Relational approach

CHWs described using a variety of practical and relational tools to build trust with a PPSD, which required a nuanced understanding of the local context and power relations. Relationships had typically been developed through multiple visits over many months and the ‘thickness’ of relationships was evident for example, Lalita’s description of Deepak’s family outlined above and her description of the range and quality of different relationships of Deepak without reference to any notes:

“Deepak travels a lot for work, he has been working to sell semi-precious stones and astrology for the last few years. This is his main income ( ) he’s married. They are expecting their first child. ( ) But because of travelling a lot he sometimes feels lonely and then he befriends others who are also travelling for work. ( ) He has described how he feels peer pressure from them to use mind altering substances. ( ) and then he also has unstable relationships with his family members and household.”

CHWs described that trust building happens over a long time period through multiple communications which facilitates a tailored response. In one situation, the CHW connected with a village head (known as a *pradhan)* who had an alcohol overuse problem. The process of building this relationship illustrated the practical and relational tools used, E.g., sharing a brochure, discussing strategies to reduce use, active listening and problem solving to build a relationship of trust as described below:

“Through our regular meetings I listened well and held dignified and respectful encounters. Then we developed action plans which Sanjay could do in the next week. So (using these) he could chart his progress and see that, and at same time he felt trustworthy and could complete his plans. A key part of this was our relationship in his house (and where we) showed told him how well he was doing.”

A further approach that improved outcomes, included supporting PPSD to build relationships with peers as a CHW working with a young man described in the quote below:

“Through an increased number of peer friendships and being a group member, I think Sunil’s self-image improved. He told me “Mujhe bahut izzit milte hai jab session chalet hai.” (I feel respected when I attend the sessions).

## Discussion

In this study, we identified particular constellations of the skills and strategies that CHWs use to engage with the ‘social’ of mental health. These include (1) deep knowledge of context and social factors impacting people with psychosocial disability which informs action on social and structural determinants of health, (2) relationships and relationality which supports outcomes and (3) delivery of tailored and bespoke care with micro-adaptations linked to their knowledge of the PPSD and family dynamics. These three factors mean that CHWs are able to address social dimensions of mental health in diverse and bespoke ways that are adapted to the specific contexts of the lives of the families and individuals with which they work.

### Relationality and outcomes

The strong relationships between CHWs and PPSD emerged from this study as a key mechanism supporting outcomes. Positive relationships have been identified as core to mental health recovery. Recently theorised approaches to relational care in health services suggest that relationships can have causal effects when enabled by the following conditions: firstly, each person has a personal and social identity known to the other. The fact that CHWs are well known and live in or near the communities they work means that they are known and can use their personal experiences to facilitate the relationship [26]. This close relationality brings important challenges. Chase [11] drawing on ethnographic research in Nepal explores what happens in the context of task-shifting when the boundaries between CHWs and community members are fluid. This fluidity was seen in our study of the bespoke and responsive care provided by CHWs, where they described unique approaches with each client that engaged with their circumstances and were less systematised. For example, focussing on self-image with Ravi, and supporting Deepak in strategies to say ‘no’ to substances with peers. Secondly, relational care is primarily characterised by caring rather than a focus on activity or performance (outputs and outcomes). This was evident in this study, for example, when the CHW describes Sanjay feeling affirmed due to the relationship rather than perceiving the relationship as transactional or conditional on performance. Thirdly, CHWs described how trust grew with a growing duration of the relationship, as described in the example with Sanjay, the village leader with alcohol use problems.

At the same time, there were problems in the investment and proximity of CHWs in relationships. CHWs were far from impartial in their relationships, and in multiple instances they showed negative judgement of men drinking alcohol, talked of persuading men to reduce their alcohol use and were frequently observed to be ‘siding’ with the women in the households of substance abusing men. This concurs with Chase’s observations that the practice of CHW can be “judgement laden and directive” and that CHWs can often use personalised over professional approaches [11]: pp. 8).

### Tailored care and cultural adaptation

This study illustrates examples of ways that CHWs offer care explicitly tailored to local contexts. Evidence-based practices developed in one place are often not appropriate, feasible or effective in a different context [21]. A large number of recent publications have identified the importance of a socially and culturally informed approach to community mental health that engages with the local context to develop endogenous solutions [4, 21]. Our dataset had various illustrations of how CHWs creatively adapted their approaches to account for local contextual and cultural aspects. For example, CHWs tailored their interventions and relationship building to account for local context and power relations within communities. Chase [9] discusses this in relation to lay psychosocial counsellors in Nepal. She illustrates how these workers flexibly use the concept of ‘psychosocial’ in their practice. For example, she discusses how CHWs developed symptom lists and descriptions that were flexibly tailored to social contexts and individual histories. This was in contrast to publicly available materials describing complaints that psychosocial counsellors could address.

Kirmayer and Pedersen [21] discuss four ways that culture contributes to social health determinants and in this study we identified two of these in CHW interactions. First, they identify the importance of “categories of identity that disadvantage specific groups”. CHWs in this study identified and addressed name-calling using their local knowledge to respond via the cultural beliefs and drivers for stigma and discrimination. Second, Kirmayer and Pedersen [21] argue that culture may provide systems of understanding that may worsen or moderate particular hardships. CHWs in this study supported the development of peer relationships and activities that shifted understanding of poverty and social exclusion for Rita (by joining the sewing group). At the same time, the solutions the CHWs proposed were conforming and non-transformational, and fully proscribed by the dominant cultural and gender relations, for example, the only suggestions CHWs provided for income generation for women with mental health problems was to work in sewing or in a beauty parlour.

### Engaging with social and structural determinants of mental health

CHWs in this study described that they supported mental health by engaging with families and communities to act on structural health determinants such as access to clean drinking water and sanitation or income generation which built on their in-depth knowledge of the context as well as skills in accessing government entitlements. A recent scoping review [2] described how CHWs can increase equitable health outcomes in LMIC, through advocacy for social and structural transformation. This review also found that CHW in particular gave additional time and resources to families who were most disadvantaged, recognising that they had fewer resources and supports. This approach suggested that CHWs were more successful in increasing equity in service delivery than in health outcomes. Further research is needed to examine whether or how CHWs support equity.

One of the counter-arguments to the approaches that engage with the ‘social’ outlined above is that these bespoke approaches do not entirely materialize just from the CHWs and their interactions. They are likely to be linked to the ‘altruistic personalities’ described in Allen and Rushton [3] among individual CHWs who may be more likely to be compassionate, pro-social and socially connected than other community members. The values, shaped through the training and ethos of the host organisation, are also likely to inform what CHWs do and how they do it [22]. There is additionally, something that happens in the interactions with PPSD that might be adaptive or bespoke. One potential way of looking at this is to conceptualize the approaches in terms of ‘thickness’ of interactions, and a layering process that takes place with different elements playing roles in a bricolage way. Additional methods which include ethnography are likely to be required to untangle and understand in-depth how CHWs develop contextualised micro-adaptations that support improved mental health outcomes among PPSD.

The micro-innovations shown by CHWs in this study are non-linear and thus dependent on elements we have identified above including local knowledge, time, and relationships. For example, when the CHW role played conversations to build Rita’s social confidence and social connection (the sewing group) and to support Rita to pursue further education. A recent review showed that in addition to the health system and economic factors, the key community-level factors that influenced CHW performance and operations included socio-cultural factors such as gender norms and values, perceived safety and security as well as the education and knowledge level of the target group [22]. In considering which of these elements could be scaled, and how in thinking about social interventions, further attentive qualitative research is needed to examine in greater depth the particular interventions or particular ways of being employed by community health workers.

### Methodological considerations

This study sourced data from community health workers active in a regional community mental health programme set in Uttarakhand, North India. The process of using cases that were randomly selected by 46 PPSD for qualitative interviews provides a breadth of identities as well as ensures a mix of eventual outcomes. A limitation of the study is that we did not directly interview PPSD meaning our findings primarily rely on CHWs’ interpretations of what happened in a given situation. This was mitigated somewhat by both KM and PP triangulating findings from CHW interviews with participant observation over several weeks of shadowing CHWs. Both PPSD, family and community members may have interpretations that differ substantially. This points to the value of future ethnographic research examining the interrelationships between different stakeholders in these care processes.

The female identity of data collectors (KM and PP) as well as all women CHWs is likely to have influenced responses by CHWs, and may have limited representation of the approaches used by men CHWs and men PPSD.

Transferability to other contexts was given attention with the inclusion of a wide range of PPSD and was also strengthened with the use of case studies which provided thick description of CHW-client interactions. Although some of the findings may be applicable to other community-based mental health programs in South Asia, the context of Northern India is unique and therefore generalizations from this research should be examined alongside context descriptions. Lastly, dependability and confirmability were addressed by reflexive discussions between researchers, CHW participants, and notes on the research process, ensuring consistency across data collection and analysis processes.

## Conclusion

Addressing social aspects of mental health is key to the approaches used by community mental health workers in this study set in low-income urban North India. Although CHWs in this study worked individually with PPSD, they engaged with factors influencing access to care, as well as the wider household, with social connections and networks, and the wider context, such as income generation. Relational care that was two-way and relevant, and local contextual knowledge and skills emerged as two key factors that supported social aspects of mental health care and formed the basis of be-spoke social micro-innovations that optimised mental health outcomes.

## Footnotes

1 India’s Right to Information Act 2005 requires Government to respond to community submissions about the rights and access to services of the Government.
